# COVID-19 pandemic and the judicialization of health care: an explanatory case study^*^

**DOI:** 10.1590/1518-8345.4584.3354

**Published:** 2020-08-10

**Authors:** Eloá Carneiro Carvalho, Pedro Hugo Dantas de Oliveira Souza, Thereza Christina Mó y Mó Loureiro Varella, Norma Valéria Dantas de Oliveira Souza, Sheila Nascimento Pereira de Farias, Samira Silva Santos Soares

**Affiliations:** 1 Universidade do Estado do Rio de Janeiro, Faculdade de Enfermagem, Rio de Janeiro, RJ, Brazil.; 2 Petróleo Brasileiro S/A, Rio de Janeiro, Rio de Janeiro, RJ, Brazil.; 3 Universidade Federal do Rio de Janeiro, Escola de Enfermagem Anna Nery, Rio de Janeiro, RJ, Brazil.

**Keywords:** Health’s Judicialization, Right to Health, Coronavirus, Unified Health System, Public Policy, Nursing, Judicialización de la Salud, Derecho a la Salud, Coronavirus, Sistema Único de Salud, Política Pública, Enfermería, Judicialização da Saúde, Direito à Saúde, Coronavírus, Sistema Único de Saúde, Política Pública, Enfermagem

## Abstract

**Objective:**

to identify the reasons that led to the judicialization of health care in the context of the COVID-19 pandemic; describe the outcomes of lawsuits concerning health care involving the COVID-19; and analyze the cases of health care judicialization intended to ensure the population’s right to health.

**Method:**

qualitative, explanatory case study. Data were collected from the websites of the Federal Prosecution Service, Regional Labor Court (1^st^ Region), and the Court of Justice of Rio de Janeiro. The inclusion criterion was public civil actions that concerned health care and situations involving the COVID-19 pandemic. Two categories emerged from data analysis.

**Results:**

four cases were identified.

**Conclusion:**

the judicialization of health care consists of obtaining assets and rights in the courts. These assets and rights are essential to ensure the health of citizens but have been denied in various instances, often due to the omission of the executive and legislative powers. Analyzing the judicialization of health care amidst the pandemic brings focus and highlights the importance of giving voice and visibility to the enormous contingent of the Brazilian society unassisted by public authorities.

## Abstract (es)

**Objetivo:**

determinar las razones que han llevado a la judicialización de la salud en relación con la pandemia de la Covid-19, describir el resultado de las acciones judiciales en materia de atención sanitaria que abarcan la Covid-19 y analizar los casos de judicialización de la salud relacionados con la pandemia de Covid-19 con vistas a garantizar el derecho a la salud de la población.

**Método:**

cualitativo, del tipo estudio de caso explicativo. Los datos fueron recogidos en los sitios web de la Fiscalía Pública, del Tribunal Regional del Trabajo (1ª Región) y del Tribunal de Justicia del Estado de Río de Janeiro. Para cumplir con los criterios establecidos, se seleccionaron las acciones civiles públicas relacionadas con la salud y la pandemia de Covid-19 con decisiones. El análisis de los datos culminó en la elaboración de dos categorías.

**Resultados:**

fueron captados cuatro casos.

**Conclusión:**

la judicialización de la salud consiste en obtener bienes y derechos en los tribunales, que son importantes para la garantía de la salud del ciudadano que, en diversas situaciones, ha sido negada, generalmente por la omisión de los poderes ejecutivo y legislativo. Con la pandemia, analizar la judicialización podría poner de relieve y plantear al debate la importancia de dar voz y visibilidad al enorme contingente de la sociedad brasileña que no es asistido por el poder público.

## Abstract (pt)

**Objetivo:**

identificar as razões que conduziram à judicialização da saúde relativas à pandemia da COVID-19, descrever o desfecho das ações judiciais relacionadas à assistência em saúde abrangendo a COVID-19 e analisar os casos de judicialização da saúde relacionados à pandemia da COVID-19 com vistas à garantia do direito à saúde da população.

**Método:**

trata-se de um estudo qualitativo, do tipo estudo de caso explicativo. A coleta foi efetuada nos *sites* do Ministério Público Federal, do Tribunal Regional do Trabalho (1ª Região) e do Tribunal de Justiça do Estado do Rio de Janeiro. Para atender aos critérios estabelecidos, selecionaram-se as ações civis públicas relacionadas à saúde e às situações da pandemia da COVID-19 com decisões. A análise dos dados culminou na elaboração de duas categorias.

**Resultados:**

captaram-se quatro casos.

**Conclusão:**

a judicialização na saúde consiste em obter bens e direitos nos tribunais, os quais são importantes para a garantia da saúde do cidadão que, em diversas situações, vem sendo negada, geralmente pela omissão dos poderes Executivo e Legislativo. Com a pandemia, analisar a judicialização poderá colocar foco e trazer à discussão a importância de dar voz e visibilidade ao enorme contingente da sociedade brasileira que não é assistido pelo Poder Público.

## Introduction

Health is generally conceived as the greatest asset of human beings. Such understanding is based on the fact that, in the absence of health, one cannot remain active and functioning in society.

The concept of health involves objective and subjective aspects. In this sense, it is characterized as a balance between human beings and the environment, enabling people to play their social, familial and occupational roles, a situation in which physical, biological and psychosocial aggressors are either contained or eliminated^(1)^.

From the perspective of its objectivity, health is the expression of quality of life, resulting from one’s eating habits, housing, education, income, environment, work, transportation, employment, leisure, freedom, access to and possession of land, and access to health services. Thus, the results of the social organization of production may generate inequalities in terms of living standards, leading to illness, sequelae, and even death^(2)^.

The health of individuals is an essential condition for society to keep growing, developing, and progressing. In this sense, healthy life requires more than people complying with their responsibilities at an individual level; it also requires protection from the part of public authorities.

In Brazil, the Federal Constitution (FC) from 1988 incorporated the recommendations of the Brazilian health movement that resulted from extensive discussions with the organized civil society. The Section dealing with health contains the entrenched clause, Article 196 in which “health is the right of all and a duty of the State”^(3)^, and establishes how the health system is organized and financed.

Based on constitutional principles, the Brazilian Unified Health System (SUS) is responsible for promoting health, preventing and curing diseases, and promoting the rehabilitation of people, while the private sector can provide complementary health services. The private sector, however, assumed new contours and arrangements that resulted on it providing Supplementary health services^(4)^.

The SUS, regulated by Laws No. 8080/1990 and No. 8.142/1990, is one of the largest and most complex public health systems in the world, the ideological principles of which are universality, integrality, and equity for the entire population. It involves the three levels of the Federation: Union, States and Cities. The network composing the SUS encompasses primary, secondary, tertiary and quaternary care; urgent and emergency services, hospital care, and epidemiological, health and environmental surveillance actions, in addition to pharmaceutical care^(5)^.

Supplementary healthcare is regulated by Law No. 9.656 from 1998, which determined the creation of the *Agência Nacional de Saúde Suplementar* (ANS) [National Supplementary Health Agency]. Supplementary healthcare is an alternative for people to obtain health services, involving the operation of private care plans and insurances^(4)^.

This sector is regulated by the government through its regulating agency – ANS and operators comprise specialized health insurers, group medicines, cooperatives, philanthropic and self-managed institutions, which in turn, demand financial payment, that is, affiliates have to pay for the services^(4)^.

Note that, due to different reasons, both the public and private health systems present problems in the delivery of healthcare, problems that compromise the quality of care delivery and lead users to become dissatisfied.

At the public level, problems involve the precariousness and dismantling of physical structures; lack of material and human resources; and a decreased number of health units, hindering the access of the population to diagnostic and therapeutic methods. These problems originate from liberal ideas implemented in work organizations, the most devastating principle of which is the insufficient dimensioning of the public structure, transferring fewer and fewer funds that would enable its functioning, restricting or making public tenders unfeasible^(6)^. Such a context tends to worsen with the Constitutional Amendment Proposal No. 55 from 2016, which prevents investments in health and education for 20 years^(7)^.

At the same time, social inequality, economic recession, new forms in which work is organized and explored in the Brazilian society have increasingly put pressure on SUS. There are two ends that do not meet: on the one hand, there is a system that lacks financing and is vilified by inconsequential governments, and on the other hand, there are growing misery and illnesses^(8)^.

Therefore, negative unfolding impacts the quality of care delivery, causing suffering to people and worsening their health conditions, leading to dissatisfaction and causing psychophysical illnesses among workers, as well as absenteeism and presenteeism^(9)^.

Supplementary healthcare services also present problems, involving increasingly higher financial payments, with often abusive adjustments of prices, without, however, providing services that correspond to the increase in monthly fees. Additionally, there are frauds, an excessive number of exams, and unnecessary procedures. At the same time, health workers, especially non-physicians, are dissatisfied with salaries that do not keep up with inflation, undersized staff, pressure, and increased demand for productivity and the need to meet increasingly higher goals^(4)^.

Such a context compromises the quality of services and results in ill workers, who experience psychological distress due to hierarchical relationships permeated by moral harassment that cause fear, while not fully meeting the health needs of patients^(10)^.

Therefore, the public and private sectors present contexts that negatively impact and prevent the healthy progress of actions developed within SUS and the supply of services to meet the health demands of the Brazilian population. Therefore, these services are subject to legal actions, so that both patients and workers have their rights fulfilled, according to what is provided by the Constitution and complementary laws^(11)^.

The COVID-19 pandemic has bluntly revealed social ills and health system problems. The collapse of the latter, due to the growing number of people infected and due to the aggressiveness nature of the SARS-CoV-2, is already a reality for some Brazilian states, while other states are heading towards it. The demand for complex care, the use of diverse technologies, an insufficient number of personal protective equipment (PPE), lack of hospitalization beds, and insufficient quality and number of health workers explain the dire situation health services face^(12-13)^.

The population and workers alike will grow dissatisfied with the Brazilian health system and its working conditions. From this perspective, the judicialization of health care is likely to magnify during the epidemic.

Such reflections and concerns encouraged this paper, the object of which deals with the judicialization of health care related to COVID-19 cases. The following problem was selected: what are the reasons and outcomes of the judicialization of cases related to COVID-19, as well as the impacts on the fulfillment of the fundamental right to health?

This study’s objective is threefold: i) to identify the reasons that led to the judicialization of healthcare related to the COVID-19 pandemic; ii) to describe the outcome of lawsuits related to care delivery concerning COVID-19; and iii) to analyze judicialization cases related to the COVID-19 pandemic in order to ensure the population’s right to health.

## Method

This is a qualitative, exploratory case study. Case studies are intended to explain, explore or describe current phenomena inserted in their own context. Thus, it is appropriate to understand how and the reasons that led to certain decisions^(14-15)^.

Secondary data were collected, specifically from the sites of the Federal Prosecution Service, Regional Labor Court – 1^st^ Region (Rio de Janeiro) and the Court of Justice of the state of Rio de Janeiro, which are public and, therefore, dispense institutional review board approval. Additionally, information was collected from documents related to laws and legal decisions.

The option to investigate the cases that took place in the state of Rio de Janeiro was based on the following: i) Rio de Janeiro is one of the states most severely affected by the epidemic; ii) it was the first to adopt social isolation measures; and iii) the phenomenon was identified by researchers in the region.

Data were collected in April 2020 using a form addressing the following information: places where lawsuits were filed; dates on which lawsuits were filed; case numbers; reasons justifying the lawsuits; parties involved in the court lawsuits; and respective outcomes.

The cases were chosen based on the following criteria: being public civil actions related to health and situations related to the COVID-19 pandemic, which contained decisions, albeit of a precarious nature, considering it was a preliminary (not definitive) pronouncement. Four cases met this criterion.

Data analysis was based on the following procedures: synthetic description of selected cases, focusing on content that enabled understanding the problem; comparison of content of legal decisions according to law and jurisprudence; and the establishment of two analytical categories that permitted discussing the cases in the light of the literature.

## Results

###  Case 1 – Process No. 0084141-46.2020.8.19.0001 

On April 22^nd^, 2020, the Public Prosecutors Office of the state of Rio de Janeiro (MPRJ) filed a public civil action against the City Hall of Barra Mansa, RJ because, on April 21^st^, 2020 it announced on social media the relaxation of social isolation measures, including the reopening of the local business starting on April 27^th^, 2020.

In addition to this fact, one should consider the situation of this city’s health system, that is, the number of Intensive Care Unit (ICU) beds and mechanical ventilators in the city and in the region’s referral hospital was insufficient to support a decision to relax the social isolation.

Therefore, the Judicial Power had to intervene in order to avoid the relaxation of social isolation measures in the city and prevent the dissemination of the virus in the region. Hence, urgent protection was requested, determining social restriction measures to remain.

Even though the judge of the Judicial Notary Office 7 – Volta Redonda and adjacent areas, considered both the citizens’ right to health and economic issues, as the suspension of economic activities for extended periods may harm the economy, she accepted the arguments presented by the Public Prosecutors Office to *grant* urgent protection in order to keep social isolation measures in the city of Barra Mansa-RJ.

The judge understood that the stir involving the disease lies in the fact that it causes an exponential increase in the demand for health services (it spreads very easily and rapidly), so that the public system – whether in its public or private branch – is not capable to provide appropriate care, as it would involve not only consultations and medical diagnoses, but also ICU hospitalization for the most severe cases with the provision of mechanical ventilators, which are currently very scarce.

###  Case 2 – Process No. 0100300-73.2020.5.01.0047 

The Rio de Janeiro Nurses Union filed a public civil action in the Labor Court (1^st^ Region – Rio de Janeiro) on April 13^th^, 2020, to obtain a court order for nurses providing care in the health services of the city of Rio de Janeiro who belonged to risk groups, to be liberated and allowed to work in a home office regime during the pandemic.

The lawsuit was filed because the city, through the General Coordination of Human Resources, issued an Official Notice (CVL/SUBSC/CGRH/CTNRH No. 04/2020) on March 18^th^, 2020, establishing the rules to release employees from work due to the coronavirus, but had not, up to that time, commented on the employees hired by social organizations to provide health care in the city.

The union argued that these workers are more likely to progress to a more severe condition due to the infection, considering they are in direct and continuous contact with infected patients and exposed to risks. It also claimed there was a lack of PPE, and for this reason, many health workers had been contaminated with COVID-19, exponentially raising the risk of illness.

In this context, the Judge of the 47^th^ Labor Court of Rio de Janeiro, granted on April 20^th^, 2020, the request for urgent relief requested by the Union to grant the interruption of provision of services on the part of health workers without interrupting the payment of their salaries.

The judge based his decision on Articles 5^th^ and 230^th^ of CF/88, which ensure that the right to life and human dignity is a duty of the state. Unsatisfied, the state of Rio de Janeiro and *Fundação Saúde* [Health Foundation] requested the order to be suspended in court. Among the reasons provided, it stand out that the decision did not take into account the multiple aspects that accrue from the absence of health workers belonging to risk groups from work that harm the health system.

In this context, they argued, based on technical precepts, that most of those involved in the combat against the COVID-19 pandemic are health workers older than 60 years old, with professional experience and occupying strategic positions. They also note that the provisional emergency protection interferes with public health policies implemented by the Executive Power, interfering in its autonomy.

Thus, the Chief Justice of the Regional Labor Court - 1^st^ Region decided to suspend the decision of the judge of the first instance court. Consequently, the nurses belonging to risk groups should return to work. The decision was based on the impact it would cause, such as a decrease in the number of nurses, which would impair the delivery of care.

Additionally, Decree No. 10.282/2020, regulated the essential activities for the purposes set out in Law No. 13.979/2020, providing for measures dealing with the public health emergency accruing from COVID-19, establishing that health care services are essential to meet the needs of the population.

###  Case 3: Process No. 42.2020.8.19.0001 

The Public Prosecutors Office and Public Defense from the state of Rio de Janeiro filed, on April 17^th^, 2020, a public civil action against the city and state of Rio de Janeiro, aiming to obtain a preliminary order to unblock all the beds reserved for ICUs and Severe Acute Respiratory Syndrome (ICU/SRAG) in the state and city of Rio de Janeiro, as provided in the State Contingency Plan – except those in Field Hospitals (scheduled to open on April 30^th^, 2020).

The lawsuit was filed because the Public Prosecutors Office, in consultation with the National Regulation System, verified that a portion of the ICU/SRAG beds, which are considered necessary to provide care to patients suspected and infected with COVID-19, was not yet effectively available.

The argument was that, even though both the state and city of Rio de Janeiro had previously developed a contingency plan, it had not been effectively implemented. Additionally, they warned about the factors increasing the demand, such as the fact that the city of Rio de Janeiro has a historical deficit of 263 adult ICU beds; the possibility of insufficient ICU/SRAG beds and, as a consequence, that the health system would collapse before the field hospitals became available (scheduled to open on April 30^th^, 2020); the exponential increase (from three cases in March to 11 at the beginning of April) of individual demands in the defense office, aimed at obtaining emergency access to ICU beds by citizens suspected or confirmed of having COVID-19.

In the first instance court, the judge of the 14^th^ Public Finance Court dismissed the preliminary request based on the argument that an intervention on the part of the judicial branch would offend the principle of separation of powers (the judiciary cannot interfere in political decisions of the other powers, except in exceptional cases), also considering the principle of possible reserve (i.e., as the budget is finite, managers, based on technical evidence, make political decisions to allocate resources the best way possible). Additionally, he maintained that other diseases could not be ignored.

The Public Prosecutors Office appealed the decision, and upon arriving at the second instance, the judge of the Second Chamber decided to comply with the request, determining that the state and city put all the ICU/SRAG beds into operation within five days, in accordance to the State Contingency Plan, with exception of those reserved for the Field Hospitals (with opening scheduled for April 30^th^, 2020).

The judge also determined that all material and human resources necessary to fulfill and enable immediate functioning would be organized. He argued that the beds were not reserved for other purposes and the federated entities were not complying with the commitment they assumed to implement the Contingency Plans and that the principle of possible reserve did not apply in this case, considering that the federated entities committed themselves to implement health measures and to create the ICU/SRAG beds indicated in the Contingency Plan of the state of Rio de Janeiro.

###  Case 4: Process No. 64.2020.8.19.0001 

This public civil action was issued by the Public Prosecution Office of Rio de Janeiro, against the state and city of Rio de Janeiro, on April 8^th^, 2020, to obtain an order to prevent the dissemination of the new coronavirus in Long-Stay Institutions for the Elderly (LSIEs).

The lawsuit was filed to ensure the fundamental rights to health and life of the most vulnerable population, that is, elderly individuals living in these institutions.

The Public Prosecutors Office justifies the demand because of a lack of a contingency plan intended to prevent the dissemination of the coronavirus in LSIEs, as well as an increase in the number of cases reported among elderly individuals living in LSIEs and their employees, along with a lack of medications, PPE, personal hygiene and cleaning products, as recommended by the State Department of Health, Health Surveillance and ANVISA (Brazilian Health Regulatory Agency).

In the first instance court, the judge of the 15^th^ Public Finance Court partially accepted the request and sentenced the defendants to:

- Provide a reserved place to accommodate the elderly (sheltered) individuals suspected or contaminated by SARS-CoV-2, who do not require hospitalization, as well as provide health workers, general services, support, medication, PPE, personal hygiene and cleaning products, as recommended by the resolutions and technical notes issued by the State Department of Health, Municipal Department of Health, and Sanitary Surveillance;
- Ensure a differentiated flow for the primary care provided to sheltered elderly individuals immediately after reporting the suspected cases to the Health Surveillance. Public authorities have to provide immediate care, sending a team of health and social workers to the shelter, occasion when testing must be carried out; and
- Ensure that these institutions receive PPE and essential hygiene and cleaning items as well as providing training to those working in these places, with continuous education and monitoring of their procedures.

## Discussion

### Category 1 – Application of the human dignity principle in times of pandemic

The principle of human dignity guides the entire Brazilian legal system. It is at the highest level and validates all the remaining rules. By putting human beings as the reference of the Brazilian law, the FC/88 shows that all people are endowed with fundamental rights, especially the right to Health, which should be appreciated in all analyzes^(16)^.

Therefore, when considering concrete cases, the principle of human dignity in the Brazilian state should illuminate both jurists and politicians. In this sense, it has the ability to protect individuals and their vulnerabilities, influencing legal decisions when there is conflict regarding such a subject.

This basic principle, found in Article 1^st^, item III of the 1988 Constitution, is considered one of the foundations of the democratic state governed by the rule of law, and must minimally ensure the preservation and appreciation of human life^(16)^.

Currently, there is a pandemic marked in the Brazilian context by great political instability, social disparities, unequal access to the health system^(17)^, and inappropriate working conditions (low salaries, precarious PPE, and insufficient material) faced by health workers, especially nurses^(13,18)^, which result in inadequate work organization.

This problem is mainly due to the sudden increase in the number of patients in intensive care units, which are invariably overcrowded, with an insufficient number of care devices, overloading the health system. This situation affects health workers who are dealing with exhausting working hours, negatively influencing their physiological and psychological needs, in addition to the safety at work^(9,13,18)^.

The protection of workers has been identified as a primary and strategic measure to face the pandemic. An efficient strategic plan was established in the United Kingdom to ensure workers have PPE^(19)^. In Russia, on the other hand, the lack of PPE is marked by a growing number of health workers becoming infected^(20)^.

In Brazil, insufficient PPE is a problem to be combated due to the growing number of deaths among health workers, especially nursing workers^(13,18)^. From this perspective, this scarcity results in a more significant number of infected cases and deaths, compromising the human dignity of patients and workers, who face precarious working conditions and a higher risk of death. This situation is aggravated even more with the political and economic stability Brazil is experiencing^(17)^.

To apply the principle of human dignity means to ratify the democratic rule of law; valuing this principle implies respecting human beings, whose lives are their greatest asset. In this context, lawsuits have been filed to ensure rights, especially the right to health.

Therefore, it is important to highlight the need for discussing the judicialization of healthcare, addressing two contents: right and health, having as framework social and economic aspects, all integral and necessary to the establishment of Public Health Policy^(11)^.

### Category 2 – Fight the pandemic as a fundamental aspect of right to health.

As for the pandemic that currently plagues the state of Rio de Janeiro, some measures were taken as a pressing need to decrease contagion by the coronavirus, such as social isolation measures.

This status was also declared by the Federal Union as well as by the World Health Organization as National Public Health Emergency^(21)^, which reiterates the adoption of strategic measures intended to suppress or reduce the circulation of the virus, due to the expressive number of cases and compromised capacity of the state to respond to the epidemic.

Hence, the Judicial Power is in favor of keeping social isolation as a way to safeguard the fundamental right to health, considering it one of the main measures to combat the virus.

At the same time, the fragility of the SUS in the face of the universalization of health services, the need to equally include the entire population in the system, with an emphasis on the performance of highly complex procedures, and ultimately, the use of ICU beds, which are expensive equipment with high technological density, becomes apparent. The relationship between demand and supply is compromised, generating long waiting lines^(22)^.

Due to the worsening of the epidemiological expectation in Brazil for the coming weeks and months, it is necessary that extraordinary measures be adopted in the context of the pandemic. One of these measures is to break the barrier between the private and public sectors at a hospital level, especially to make beds available, improving the capacity to respond to the situation of calamity.

Hence, the Ministry of Health, the city and state Departments and regulatory agencies need to urgently standardize national regulations regarding access to beds in the exceptional case of the pandemic. Note that it is vital that a single line for ICU beds is created for the severe cases of COVID-19 due to the public, transitory and urgent need, in order to prevent hospitals keep beds idle, and ensure the fundamental right to health. This proposition is based on Article 5^th^, item XXV of the FC/988, and law 8080/1990, in its Article 15, item XIII^(23)^.

Another aspect refers to the imminent risk of contagion and the growing need of the population for health care, a situation in which nurses need to work despite a pre-existing precarious context at all levels of health care, which has become increasingly acute as the numbers of COVID-19 cases increase.

The Brazilian Federal Council of Nursing (Cofen) recommends that nursing workers belonging to the risk groups avoid working at the front line to fight COVID-19. The reason is that nursing workers are in direct contact with infected individuals, which increases the risk of potential contagion. The situation worsens with the Provisional Measure 927/2020, issued by the President of the Republic, which allows the extension of working hours and the reduction of mandatory rest for these workers^(24)^.

Note that in Brazil, according to Cofen, 98 nursing workers had died by May 7^th^, 2020, surpassing the number of deaths in the United States where, according to the National Nurses United, a total of 91 nursing workers had died. According to the International Council of Nurses (ICN), 260 workers have lost their lives in the service of saving others worldwide^(17)^.

Therefore, in order to ensure the right to health and life for these workers, allowing those who belong to risk groups to be absent from work is a sound measure that should be standardized and adopted in the entire country.

In Brazil, the adoption of measures is not uniform. In Espírito Santo, for instance, a technical note was issued that recommended that nursing workers consider leaving work, however, when not possible, preferably be assigned to administrative tasks rather than directly providing care to patients infected with coronavirus^(25)^.

Therefore, adopting protective and work organization that includes preventive measures is essential in the contemporaneous context. One should continually assess the health profile of these workers because the goal is to save lives, including those of health workers, and ensure human dignity.

The COVID-19 pandemic has enabled assessing various healthcare segments in different contexts. This is a time to assess, to establish national and international cooperation, and to understand that all lives are valuable and one’s life is as important as someone else’s life^(26)^.

In this line of reasoning, thinking about the elderly is urgent, considering that the virus is more lethal among those of advanced age. Hence, preserving life and thinking about those living in collective spaces is extremely important and urgent in order to ensure the health and protection of those living in long-stay institutions.

In Brazil, an LSIE is defined by ANVISA, in Collegiate Board Resolution 283, as governmental or non-governmental institutions, the purpose of which is to shelter people aged 60 or over, granting them dignity and citizenship rights, in order to provide social and health services^(27)^.

Therefore, ensuring safe conditions to elderly individuals and workers in these spaces is essential. The provision of PPE to workers and inputs to protect the residents of LSIEs is a condition necessary to promote the constitutional right of human dignity.

We need to have focus and discipline to face the adversities accruing from SARS-CoV-2, which in Brazil are inserted in a context of social inequality, decreased investment in science (such as drastic cuts in research funding), in social security and in public services in general^(17)^.

There is an international appeal for society to invest in science, incorporating the knowledge produced in research in the public policies of countries and in international treaties^(28)^.

This study’s contributions include the analysis of concrete cases involving the judicialization of health care in the face of the COVID-19 pandemic, bringing to light measures that need to be considered and adopted to ensure the right to health. Additionally, this study presents some public civil actions filed thus far, as well as situations that require attention considering the crisis of the health sector, which demand that changes be implemented in the public and private health systems.

This study’s limitations include the fact that data were collected only in Rio de Janeiro and also that at least three or more triangulation techniques were not adopted. The reason is due to the context of the pandemic itself, which hindered access to the context of care provided to patients with COVID-19 to collect data.

## Conclusion

The analysis of the cases led to the conclusion that the objective of the judicialization of health care was to ensure that the public authorities would comply with the fundamental rights of citizens, as well as with technical and scientific recommendations and measures intended to protect the population during the pandemic.

In this sense, the synthesis of the results is linked to the need to protect the fundamental rights established in entrenched clauses of the Federal Constitution, guided by the principle of human dignity, a guiding command of the entire legal system.

Analyzing the judicialization of health care at this time of the pandemic brings to light the fact that there is a huge contingent of the Brazilian society that is not properly assisted by public authorities, highlighting the fragility of the system, the potential for the pandemic to worsen, as well as a potential increase in the number of deaths due to COVID-19.

The judicialization of health care reveals a lack of compliance with constitutional prerogatives concerning right to health and human dignity as well the state’s inability to fulfill the right of citizens who become invisible in a society marked by inequality and enormous wealth concentration.

From this perspective, the government, at its three levels, should commit to the enforcement of constitutional rules, making effective and materializing fundamental rights in order to enable health workers and the population to have a healthy life. These measures ensure not only the compliance of rights, but also evidence the ethical duty of government officials and the need of a state that is capable to promote equity.
